# Knockdown of hsa_circ_0001275 reverses dexamethasone-induced osteoblast growth inhibition via mediation of miR-377/CDKN1B axis

**DOI:** 10.1371/journal.pone.0252126

**Published:** 2021-05-27

**Authors:** Yan Xu, Liqin Sun, Juncheng Hu, Sai Xu

**Affiliations:** Department of Endocrinology, The First People’s Hospital of Fuyang District, Hangzhou, Zhejiang, China; Northwest University, UNITED STATES

## Abstract

**Background:**

Osteoporosis affects the quality of life among middle-aged and elderly individuals. In addition, dysfunction of osteoblasts can lead to the progression of osteoporosis. Circular (circ)RNAs are involved in various types of diseases, including osteoporosis. Moreover, it has been reported that hsa_circ_0001275 expression is upregulated in osteoporosis. However, the effects of hsa_circ_0001275 on the growth of osteoblasts remain unclear.

**Methods:**

In the present study, the gene and protein expression levels in hFOB1.19 cells were detected via reverse transcription-quantitative (RT-qPCR) and western blot analyses, respectively. In addition, alkaline phosphatase (ALP) activity and calcium nodules were examined by ALP and alizarin red staining, respectively. Cell proliferation was measured using the Cell Counting Kit-8 assay. Cell apoptosis and cell cycle were analyzed by flow cytometry. Furthermore, dual luciferase reporter and RNA pull-down assay were used to confirm the association among hsa_circ_0001275, microRNA (miR)-377 and CDKN1B.

**Results:**

DEX-induced hFOB1.19 cell growth inhibition was significantly reversed by silencing hsa_circ_0001275. Moreover, DEX significantly increased ALP activity and calcium nodules in hFOB1.19 cells, while this effect was significantly reversed in the presence of hsa_circ_0001275 small interfering RNA. In addition, miR-377 was sponged by hsa_circ_0001275 and CDKN1B was directly targeted by miR-377 in hFOB1.19 cells. Furthermore, the therapeutic effect of hsa_circ_0001275 knockdown on osteoporosis was notably reversed by miR-377 antagomir.

**Conclusion:**

The data demonstrated that knockdown of hsa_circ_0001275 reversed DEX-induced osteoblast growth inhibition via activation of the miR-377/CDKN1B axis. Therefore, this study might shed new lights on the treatment of osteoporosis.

## Introduction

Osteoporosis is a disease of skeletal impairment that occurs worldwide, which results in increased fragility or decreased bone mass [1]. The regulation processes of bone homeostasis correlate mainly with multiple factors, including hormones, mechanical stimulation and epigenetic regulation [2, 3]. At present, the major treatments of osteoporosis are surgery and drug therapy, while the effects remain unsatisfactory. Moreover, dysfunction of osteoblasts can lead to the progression of osteoporosis [4]. Therefore, the identification of new methods for regulating the function of osteoblasts is necessary. Meanwhile, Dexamethasone (DEX) is a synthetic glucocorticoid (GC) used widely for treating inflammatory and autoimmune diseases [5]. However, long-term DEX treatment is associated with serious adverse effects, including decreased bone mineral density and microarchitecture porosity, eventually leading to osteoporosis [6, 7]. Based on these backgrounds, osteoblasts were treated with DEX to mimic the progression of osteoporosis *in vitro* in current study.

Circular RNAs (circRNAs) are endogenous RNAs with a covalently closed cyclic structure [8, 9]. Intracellular circRNAs with competing endogenous RNA (ceRNA) activity may act as microRNA (miRNA/miR) inhibitors by interacting with miRNA recognition elements (MREs) on target mRNAs [10, 11]. This can inhibit the biological activity of miRNAs, leading to the upregulation of target genes. Based on this point, circRNAs are regarded as key mediators in multiple diseases [12, 13]. In addition, circRNAs have been reported to regulate the progression of osteoporosis [14, 15]. It has also been confirmed that hsa_circ_0001275 is upregulated in osteoporosis [16]. However, the function of hsa_circ_0001275 in osteoporosis remains unknown. Meanwhile, it has been reported that the dysfunction of osteoblasts is closely correlated with the progression of osteoporosis [17, 18]. Thus, this study sought to explore the effect of hsa_circ_0001275 on osteoblast growth, confirming whether hsa_circ_0001275 could regulate the progression of osteoporosis.

Special AT-rich sequence-binding protein 2 (SATB2) is a stemness and senescence regulator, which can regulate the progression of osteoporosis [19]. For instance, increased SATB2 has been confirmed to promote osteogenic differentiation during the progression of osteonecrosis [20, 21]; thus, upregulation of SATB2 has been reported to promote the progression of osteoporosis. On the other hand, Runx3 is a member of the Runx family of proteins, which can regulate the markers of chondrocyte maturation [22]. Zheng J *et al* found Runx3 were remarkably elevated during the progression of osteoporosis [14]; Bauer O *et al* indicated that loss of osteoblast Runx3 could produce severe congenital osteopenia [23]. Therefore, it can be suggested that Runx3 plays a crucial role in osteoporosis. Furthermore, ATF4 (activating transcription factor 4) has been identified as an osteogenic factor as it can modulate osteoporosis development. For example, ATF4 was considered as an osteogenic factor in osteoblasts [24]; ALF4 could promote the osteoblastogenesis and inhibit osteoclastogenesis during the development [25]. Collectively, these studies demonstrated that, SATB3, Runx3 and ATF4 participated in the progression of osteoporosis.

In the current study, the effects of hsa_circ_0001275 were investigated on the growth of osteoblasts. The data may provide a new strategy for the treatment of osteoporosis.

## Material and methods

### Cell culture

Osteoblasts (hFOB1.19 cell lines) were obtained from the American Type Culture Collection and cultured in DMEM (Thermo Fisher Scientific, Inc.) with 10% FBS (Thermo Fischer Scientific, Inc.) and 1% penicillin and streptomycin (Thermo Fisher Scientific, Inc.) at 37°C in the presence of 5% CO_2_.

### Cell transfection

siRNA against hsa_circ_0001275 (hsa_circ_0001275 siRNA1, hsa_circ_0001275 siRNA2 and hsa_circ_0001275 siRNA3, 10 μM) were purchased from Guangzhou RiboBio Co., Ltd. and transfected into osteoblasts using Lipofectamine^®^ 2000 (Thermo Fisher Scientific, Inc.) according to the manufacturer’s instructions. The efficiency of transfection was detected by reverse transcription-quantitative PCR (RT-qPCR). Meanwhile, the usage concentration of siRNA was 10 nM. The sequences of the siRNAs used were as follows: NC siRNA, UUCUCCGAACGUGUCACGUTT; hsa_circ_0001275 siRNA1, GGAAUGAAGCAACUGAGAUUU, hsa_circ_0001275 siRNA2, GGGTTACGATTGCCCAGAT and hsa_circ_0001275 siRNA3, ACGACCGTACCCGAACATG. hFOB1.19 cells were transfected with miR-377agomir/antagomir or negative control (NC) by Lipofectamine 2000 according to the manufacture’s protocol. miR-377 agomir, miR-377 antagomir and NC RNAs were purchased from Shanghai GenePharma Co., Ltd.

For CDKN1B overexpression, hFOB1.19 cells were transfected with pcDNA3.1 vector or pcDNA3.1-CDKN1B by Lipofectamine 2000 according to the manufacture’s protocol. pcDNA3.1 vector and pcDNA3.1-CDKN1B were obtained from Shanghai GenePharma Co., Ltd.

### RT-qPCR

Total RNA from hFOB1.19 cell lines was extracted with the TRIzol reagent (TaKaRa Bio, Inc.) according to the manufacturer’s protocol. Subsequently, Total RNA (5 μg) was reverse transcribed into cDNA using the PrimeScript RT reagent kit (ELK Biotechnology) according to the manufacturer’s protocol. The following protocol was used to perform RT-qPCR in triplicate: Initial denaturation for 2 min at 94°C, followed by 35 cycles of 30 sec at 94°C and 45 sec at 55°C. PCR were carried out using SYBR premix Ex Taq II kit (ELK Biotechnology) on an ABI 7500 Real-Time PCR system (ABI, NY, USA). The primers were obtained from Shanghai GenePharma Co., Ltd. The sequences of the primers are listed in Table 1. The 2^-ΔΔCq^ method was used to quantify the results. The internal reference gene (U6 or β-actin) was used for normalization.

**Table 1:** The sequences for primers.

Gene	Sequence of primer
Hsa_circ_0001275	Forward: 5’-TCTTCTTGAGCTAGGGCCCTT-3’
	Reverse: 5’-TGGAGTCAGAGACATGAGTGTGG-3’
CDKN1B	Forward: 5’-GGCTAACTCTGAGGACACGCA-3’
	Reverse: 5’-AGAATCGTCGGTTGCAGGTC-3’
MiR-377	Forward: 5’-TATCACACAAAGGCAACTTTTGT-3’
	Reverse: 5’-CTCAACTGGTGTCGTGGAGTC-3’
U6	Forward: 5’-CTCGCTTCGGCAGCACAT-3’
	Reverse: 5’-AACGCTTCACGAATTTGCGT-3’
β-actin	Forward: 5’-GTCCACCGCAAATGCTTCTA-3’
	Reverse: 5’-TGCTGTCACCTTCACCGTTC-3’

### CCK-8 assay

hFOB1.19 cells were seeded in 96-well plates (5x10^3^ cells per well) overnight. Subsequently, the cells were treated with 0.1, 0.5, 1 or 5 μM DEX for 48 h. The cells in each well were treated with 10 μl CCK-8 reagent and further incubated for 2 h at 37°C. Finally, the absorbance of hFOB1.19 cells was measured at 450 nm on a microplate reader (Thermo Fisher Scientific, Inc.).

### Ki-67 staining

hFOB1.19 cells were seeded in 24-well plates overnight. Subsequently, the cells were treated with DEX, DEX+hsa_circ_0001275 siRNA2 or hsa_circ_0001275 siRNA2 for 72 h. The cells were blocked with 10% goat serum for 30 min at room temperature and incubated with anti-Ki67 antibody (Abcam; 1:1,000) at 4°C overnight. Subsequently, the cells were incubated with goat anti-rabbit IgG (Abcam; 1:5,000) at 37°C for 1 h. The nuclei were stained with DAPI (Beyotime Institute of Biotechnology) for 5 min. Finally, the cells were observed under a fluorescence microscope (Olympus CX23; Olympus Corporation).

### Cell apoptosis analysis

hFOB1.19 cells were trypsinized, washed with phosphate-buffered saline and resuspended in Annexin V Binding Buffer. The cells were subsequently stained with 5 μl fluorescein isothiocyanate and 5 μl propidium iodide for 15 min. A flow cytometer (BD Biosciences) was used to determine the cell apoptosis rate.

### Dual luciferase reporter assay

The construction of the WT/MT reporter vectors for hsa_circ_0001275 and CDKN1B was performed using the partial sequences of hsa_circ_0001275 and the 3’-untranslated region (UTR) of CDKN1B containing the putative binding sites for miR-377. These sequences were synthesized by Sangon Biotech and cloned into pmirGLO Dual-Luciferase miRNA Target Expression Vectors (Promega Corporation). Lipofectamine 2000 (Thermo Fisher Scientific, Inc.) was used to transfect hFOB1.19 cells with the hsa_circ_0001275/CDKN1B (WT) or hsa_circ_0001275/CDKN1B (MT) vectors, together with the control, vector-control or miR-377 agomir, according to the manufacturer’s instructions. The relative luciferase activity was analyzed using a Dual-Glo Luciferase Assay System (Promega Corporation).

### RNA pull-down

RNA pull-down assay was performed using the biotin RNA Labeling Mix (Roche Diagnostics) in order to transcribe and label probe-control or probe-hsa_circ_0001275 from hsa_circ_0001275 siRNA2 lenti vector *in vitro*. An RNA structure buffer (Thermo Fisher Scientific, Inc.) was used to induce secondary structure formation from the biotin-labeled RNAs. Streptavidin beads (Thermo Fisher Scientific, Inc.) were washed three times with 500 μl RNA immunoprecipitation wash buffer (Thermo Fisher Scientific, Inc.) and subsequently added to the biotinylated RNAs at 4°C overnight. The overnight mixture was separated by a magnetic field so that streptavidin bead-RNA complexes could be obtained. The lysates derived from hFOB1.19 cells were added to the complexes and incubated on a rotator at room temperature for 1 h. The incubated mixture was again separated with a magnetic field so that streptavidin bead-RNA-protein complexes could be obtained. Meanwhile, the biotinylated hsa_circ_0001275 and negative control (bio-NC) were generated via Shanghai GenePharma Co., Ltd and coated to streptavidin-conjugated magnetic beads. hFOB1.19 cells were lysed and then incubated with the magnetic beads for 6 h. The RNA on the beads was isolated and the enrichment level of miR-377 was detected by RT-qPCR.

### Western blotting

Total protein was isolated from cell lysates with radio-immunoprecipitation assay buffer and quantified with a bicinchoninic acid protein assay kit (Beyotime Institute of Biotechnology). The proteins were resolved on 10% SDS and subsequently transferred to polyvinylidene difluoride membranes (Bio-Rad Laboratories, Inc.). Following blocking, the membranes were incubated with primary antibodies at 4°C overnight and subsequently incubated with an anti-rabbit secondary antibody (Abcam; 1:5,000) at room temperature for 1 h. The membranes were scanned on an Odyssey Imaging System and analyzed with Odyssey v2.0 software (LICOR Biosciences). The primary antibodies used in the present study were as follows: anti-CDKN1B (Abcam; 1:1,000), anti-SATB2 (Abcam; 1:1,000), anti-ATF4 (Abcam; 1:1,000), anti-Runx3 (Abcam; 1:1,000), anti-Cyclin E1 (Abcam; 1:1,000), anti-CDK2 (Abcam; 1:1,000), anti-Bax (Abcam; 1:1,000), anti-X-linked inhibitor of apoptosis protein (XIAP, Abcam; 1:1,000), anti-pro-caspase 3 (Abcam; 1:1,000), anti-active caspase 3 (Abcam; 1:1,000) and anti-β-actin (Abcam; 1:1,000). β-actin was used as an internal control.

### ALP staining assay

ALP staining was used to assess the ALP activity of osteoblasts. Osteoblasts were digested by trypsin and seeded in 24-well plates. Propanol (200 μl, 15 min), incubation solution (200 μl, 6 h), cobalt nitrate (200 μl, 15 min) and ammonium sulfide (200 μl, 5 min) were added successively. The optical density (OD) at 490 nm was detected using a microplate reader (Varioskan LUX; Thermo Fisher Scientific, Inc.).

### Alizarin red staining assay

The formation of calcium nodules was detected by alizarin red staining. Osteoblasts were digested and seeded in 24-well plates. Polyformaldehyde (5%, 500 μl, 10 min) and alizarin (200 μl, 37°C, 30 min) were added to the medium successively, followed by photomicrographs of calcium nodules.

### Cell cycle detection

Briefly, cell cycle detection was performed using Cycletest Plus DNA Reagent Kit (BD Biosciences). Osteoblasts were harvested by accutase treatment and counted with a hemocytometer. A total of 5x10^5^ cells were fixed, permeabilized and stained in accordance with the manufacturer’s instructions. The samples were analyzed by flow cytometry using a FACS Calibur, which measured the FL2 area signals vs. the total counts. The data were analyzed using ModFit (http://mycyte.org/) and FlowJo (http://mycyte.org/) software to generate the percentages of cells in G_1_, S and G_2_ to M phases of the cell cycle.

### Statistical analysis

Each group was assessed at least in three independent experiments and all data were expressed as the mean ± standard deviation. The differences were analyzed using the unpaired Student’s t-test (only two groups) or the one-way analysis of variance followed by the Tukey’s test (>2 groups, GraphPad Prism7). A P<0.05 was considered to indicate a statistically significant difference.

## Results

### Knockdown of hsa_circ_0001275 reverses DEX-induced inhibition of hFOB1.19 cell proliferation

**Fig 1:**
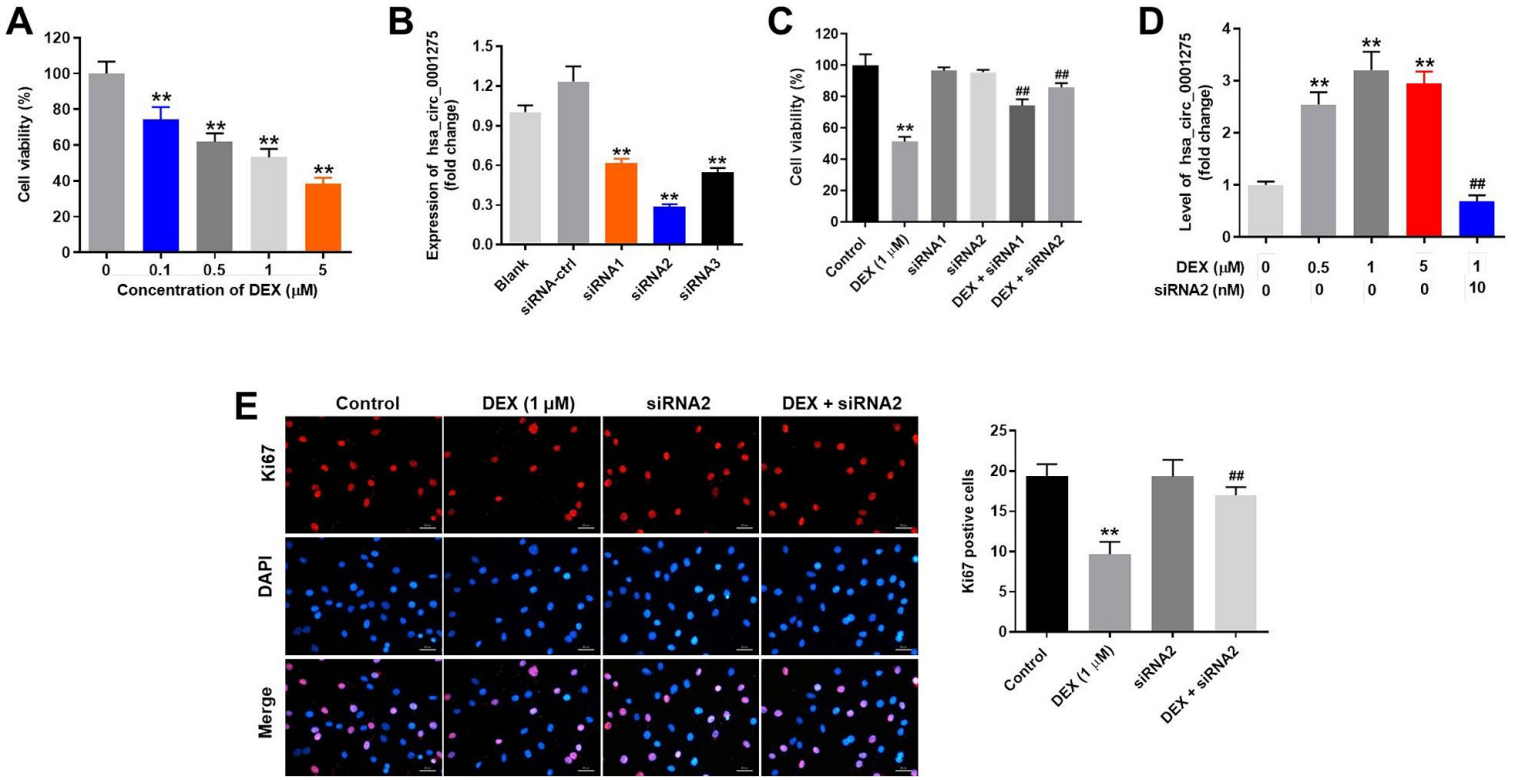
Knockdown of hsa_circ_0001275 significantly reversed DEX-induced inhibition of hFOB1.19 cell proliferation. (A) hFOB1.19 cells were treated with 0.1, 0.5, 1 or 5 μM DEX for 48 h. Then, cell viability was tested by CCK-8 assay. (B) hFOB1.19 cells were transfected with hsa_circ_0001275 siRNA1, hsa_circ_0001275 siRNA2 or hsa_circ_0001275 siRNA3. Then, the expression of hsa_circ_0001275 in hFOB1.19 cells was detected by qRT-PCR. (C) hFOB1.19 cells were treated with 1 μM DEX, hsa_circ_0001275 siRNA1, hsa_circ_0001275 siRNA2, DEX + hsa_circ_0001275 siRNA1 or DEX + hsa_circ_0001275 siRNA2. The viability of hFOB1.19 cells was tested by CCK-8 assay. (D) hFOB1.19 cells were treated with 0.5 μM DEX, 1 μM DEX, 5 μM DEX or 1μM DEX + hsa_circ_0001275 siRNA2. Then, the expression of hsa_circ_0001275 in hFOB1.19 cells was detected by qRT-PCR. (E) hFOB1.19 cells were treated with DEX or/and hsa_circ_0001275 siRNA2. Subsequently, the proliferation of hFOB1.19 cells was measured by Ki-67 staining. Red fluorescence indicates Ki-67. Blue fluorescence indicates DAPI. **P<0.01 compared to control. ^##^P<0.01 compared to 1 μM DEX.

To mimic osteoporosis *in vitro*, hFOB1.19 cells were treated with dexamethasone (DEX) as previously described [26, 27]. As indicated in Fig 1A, DEX significantly inhibited the viability of hFOB1.19 cells in a dose-dependent manner. Moreover, expression of hsa_circ_0001275 was significantly decreased in hFOB1.19 cells following transfection with hsa_circ_0001275 siRNA1/siRNA2/siRNA3 (Fig 1B), and silencing of hsa_circ_0001275 reversed DEX-induced decrease of hFOB1.19 cell viability (Fig 1C). In addition, DEX-treated hFOB1.19 cells were more sensitive to hsa_circ_0001275 siRNA2 treatment compared with hsa_circ_0001275 siRNA1 (Fig 1C). Therefore, hsa_circ_0001275 siRNA2 was selected for subsequent use in the following experiments. Furthermore, silencing of hsa_circ_0001275 reversed DEX-induced upregulation of hsa_circ_0001275 in hFOB1.19 cells (Fig 1D). Since hsa_circ_0001275 expression in hFOB1.19 cells was more susceptible to 1 μM DEX treatment, 1 μM DEX was selected for subsequent use in further experiments (Fig 1D). In addition, DEX-induced inhibition of hFOB1.19 cell proliferation was reversed by hsa_circ_0001275 siRNA2 (Fig 1E). However, hsa_circ_0001275 siRNA2 alone exhibited limited effect on cell proliferation (Fig 1E). Taken together, the data demonstrated that knockdown of hsa_circ_0001275 significantly reversed DEX-induced inhibition of hFOB1.19 cell proliferation.

### Silencing of hsa_circ_0001275 reverses DEX-induced apoptosis of hFOB1.19 cells

**Fig 2:**
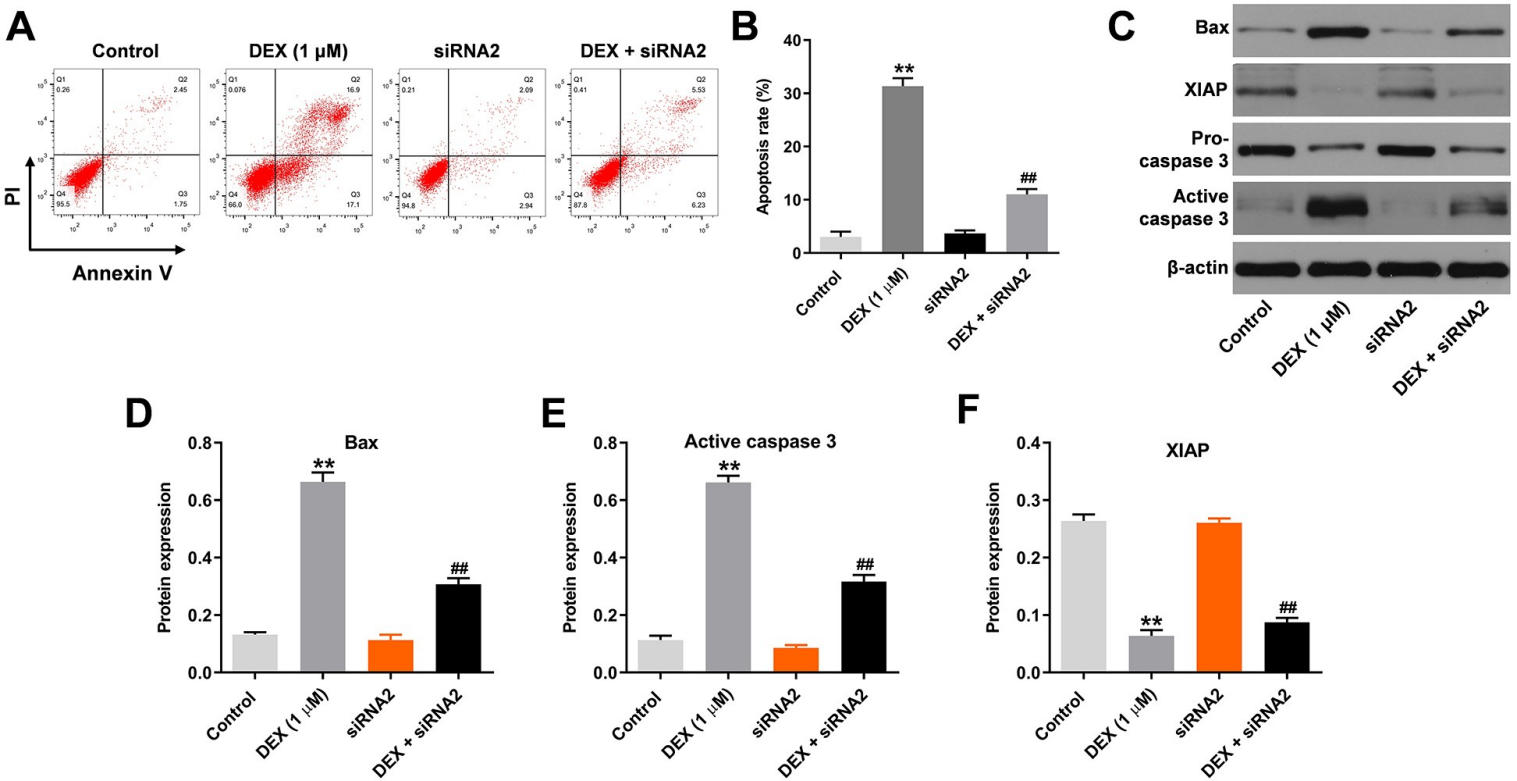
Silencing of hsa_circ_0001275reversed DEX-induced apoptosis of hFOB1.19 cells. (A) The apoptosis of hFOB1.19 cells was tested by flow cytometry. (B) The rate of apoptosis was calculated. (C) The protein expressions of Bax, XIAP, pro-caspase 3 and active caspase 3 in hFOB1.19 cells were detected by western blot. (D, E, F) The relative expressions were quantified via normalizing to β-actin. **P<0.01 compared to control. ^##^P<0.01 compared to 1 μM DEX.

In order to assess cell apoptosis, flow cytometry was performed. DEX significantly induced apoptosis of hFOB1.19 cells, while the apoptotic effect of DEX was mainly inhibited by hsa_circ_0001275 siRNA2 (Fig 2A and 2B). In addition, the expression levels of Bax and active caspase 3 in hFOB1.19 cells were significantly upregulated by DEX. These effects were significantly reversed in the presence of hsa_circ_0001275 siRNA2 (Fig 2C–2E). In contrast to these observations, DEX-induced decrease of XIAP expression was mainly rescued by hsa_circ_0001275 knockdown (Fig 2C and 2F). However, hsa_circ_0001275 siRNA2 alone exhibited a very limited effect on these three proteins (Fig 2C–2F). Collectively, the data indicated that silencing of hsa_circ_0001275 markedly inhibited DEX-induced apoptosis of hFOB1.19 cells.

### Knockdown of hsa_circ_0001275 decreased DEX-induced osteoblast mineralization

**Fig 3:**
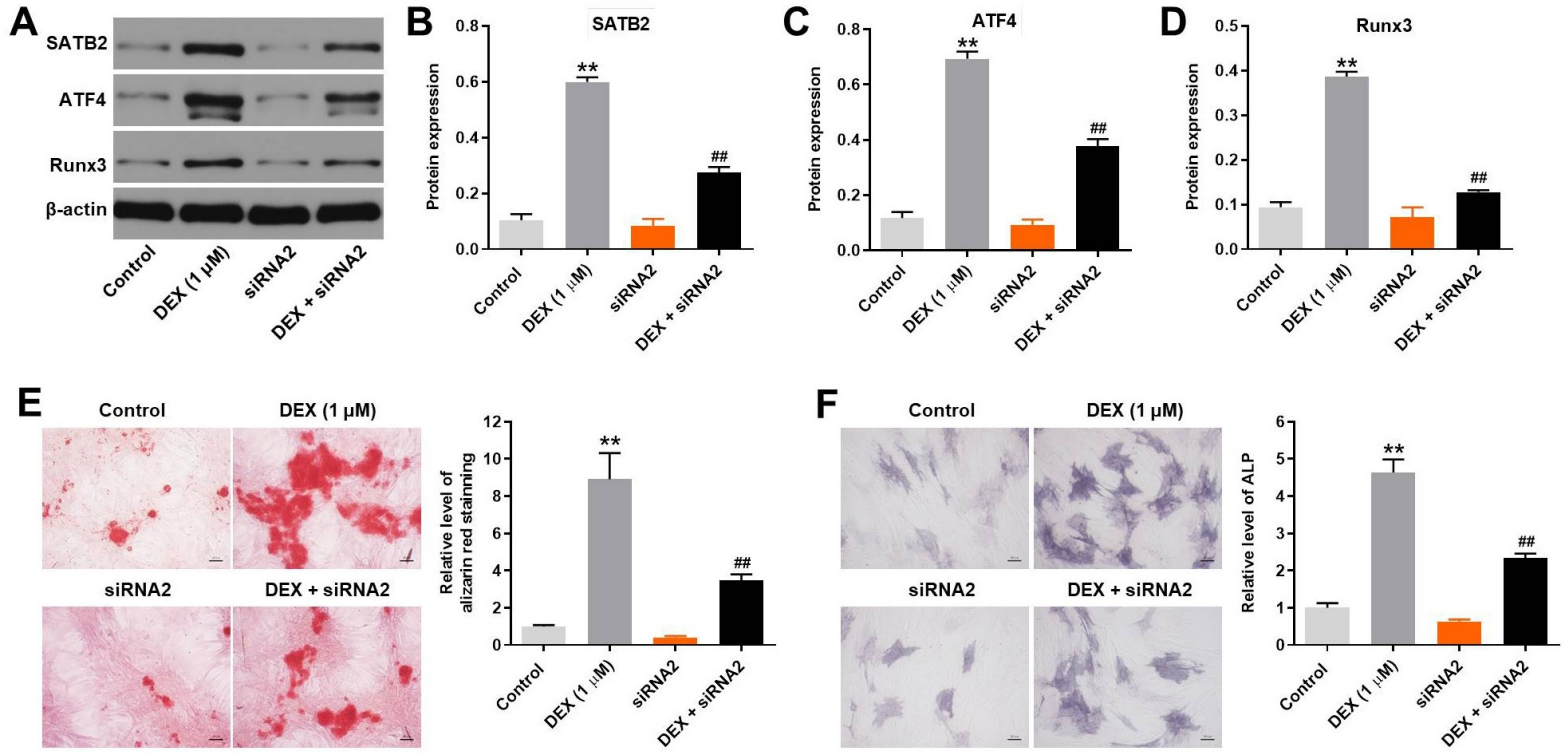
Knockdown of hsa_circ_0001275 decreased DEX-induced osteoblast mineralization. (A) The protein expressions of SATB2, ATF4 and Runx3 in hFOB1.19 cells were detected by western blot. (B, C, D) The relative expressions of SATB2, ATF4 and Runx3 were quantified via normalizing to β-actin. (E) Calcium deposits in hFOB1.19 cellswere detected by alizarin red staining. (F) The result of ALP staining in osteoblasts was presented. **P<0.01 compared to control. ^##^P<0.01 compared to 1 μM DEX.

Since SATB2, ATF4 and Runx3 are three key mediators of osteoporosis, western blot analysis was used to detect the expression levels of their proteins. As shown in Fig 3A–3D, the protein levels of SATB2, ATF4 and Runx3 in hFOB1.19 cells were upregulated by DEX treatment, which were significantly reversed in the presence of hsa_circ_0001275 siRNA2. Moreover, DEX-induced increase of calcium deposits in hFOB1.19 cells was apparently inhibited by silencing hsa_circ_0001275 (Fig 3E). The levels of ALP in hFOB1.19 cells were significantly upregulated by DEX, while the effects of DEX were significantly inhibited by hsa_circ_0001275 siRNA2 treatment (Fig 3F). In summary, knockdown of hsa_circ_0001275 decreased DEX-induced osteoblast mineralization.

### Silencing of hsa_circ_0001275 reverses DEX-induced hFOB1.19 cell growth inhibition via activation of the miR-377/CDKN1B axis

**Fig 4:**
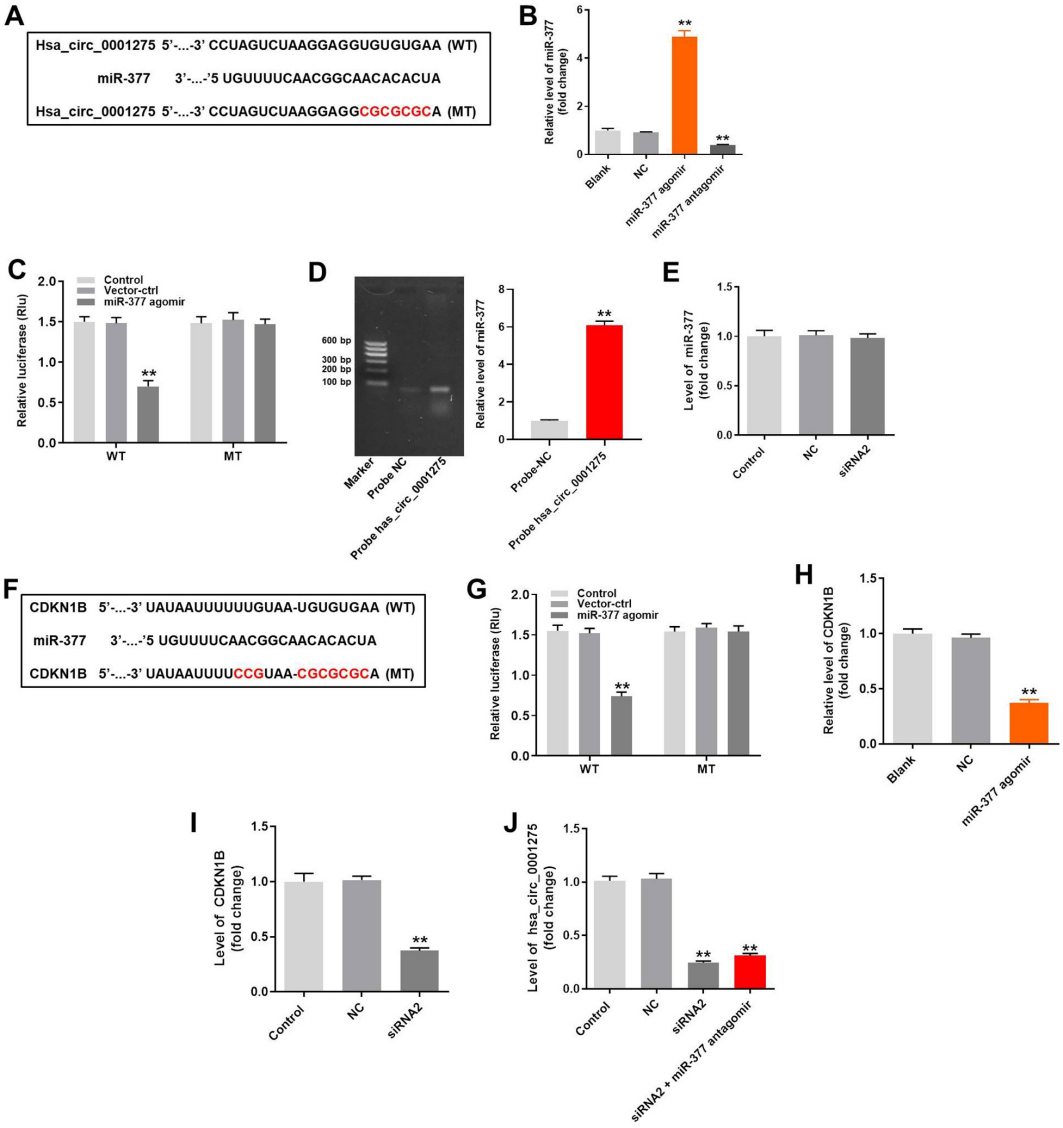
Silencing of hsa_circ_0001275 reversed DEX-induced hFOB1.19 cell growth inhibition via mediation of miR-377/CDKN1B axis. (A) Gene structure of hsa_circ_0001275 indicates the predicted target site of miR-377 in its 3’UTR. (B)hFOB1.19 cells were transfected with miR-377 agomir/antagomir for 24 h. Then, the expression of miR-377 in hFOB1.19 cells was detected by qRT-PCR. (C) The luciferase activity was measured in hFOB1.19 cells following co-transfecting with WT/MT hsa_circ_0001275 3’-UTR plasmid and miR-377 with the dual luciferase reporter assay. (D) RNA pull-down was performed to explore the correlation betweenhsa_circ_0001275 and miR-377. (E) The level of miR-377 in hFOB1.19 cells was detected by RT-qPCR. (F) Gene structure of CDKN1B indicates the predicted target site of miR-377 in its 3’UTR. (G) The luciferase activity was measured in hFOB1.19 cells following co-transfecting with WT/MT CDKN1B 3’-UTR plasmid and miR-377 with the dual luciferase reporter assay. (H, I) The expression of CDKN1B in hFOB1.19 cells was detected by RT-qPCR. (J) The level of hsa_circ_0001275 in hFOB1.19 cells was detected by RT-qPCR. **P<0.01 compared to control.

In order to identify which miRNAs were sponged by hsa_circ_0001275, the miRDB (http://www.mirdb.org/) and starbase (http://starbase.sysu.edu.cn/) databases were explored. As demonstrated in Fig 4A, hsa_circ_0001275 had a putative miR-377 targeting site, and some reports indicated that miR-377 can regulate the activity of osteoblasts [28, 29]. Thus, miR-377 was selected for further analysis. In addition, miR-377 agomir/antagomir was successfully transfected into hFOB1.19 cells (Fig 4B). Moreover, co-transfection of the wild-type hsa_circ_0001275 vector (WT-hsa_circ_0001275) with miR-377agomir reduced significantly the luciferase activities compared with those of the mutant hsa_circ_0001275 vector (MT-hsa_circ_0001275) (Fig 4C). Furthermore, the data of the RNA pull-down suggested that hsa_circ_0001275 was bound to miR-377 in hFOB1.19 cells (Fig 4D), while hsa_circ_0001275 siRNA rarely affected the level of miR-377 (Fig 4E). CDKN1B (p27 Kip1) was the direct target of miR-377 (Fig 4F and 4G). In addition, the expression levels of CDKN1B in hFOB1.19 cells were inhibited significantly by miR-377 agomir or knockdown of hsa_circ_0001275 (Fig 4H and 4I). Besides, miR-377 antagomir did not affect hsa_circ_0001275 siRNA-induced decrease of hsa_circ_0001275 expression (Fig 4J). Taken together, silencing of hsa_circ_0001275 reversed DEX-induced hFOB1.19 cell growth inhibition via activation of the miR-377/CDKN1B axis.

### miR-377 antagomir significantly reverses the effect of hsa_circ_0001275 siRNA2 on cell cycle distribution

**Fig 5:**
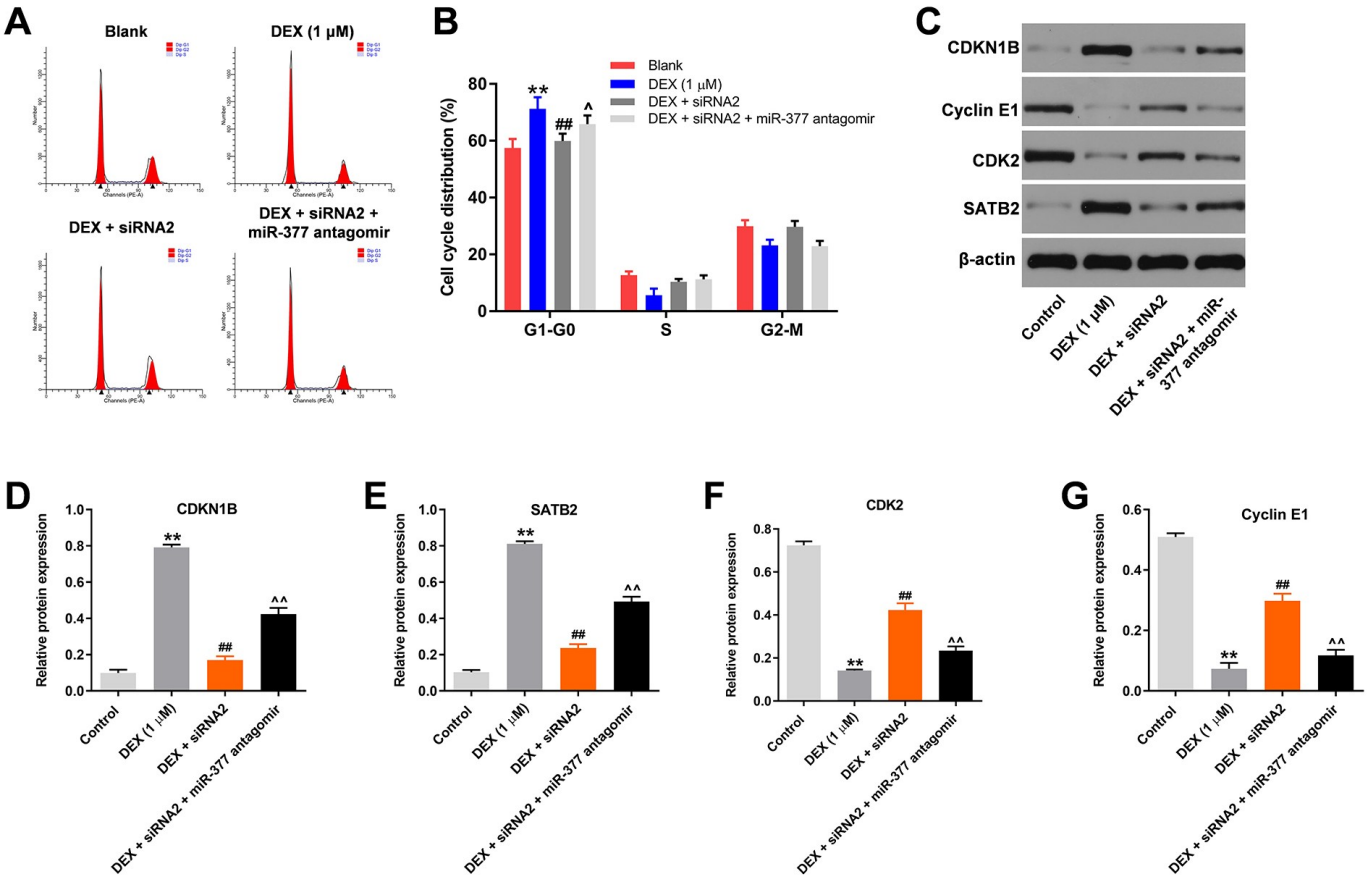
MiR-377 antagomir significantly reversed the effect of hsa_circ_0001275 siRNA2 on cell cycle distribution. (A, B) Cell cycle distribution was tested by flow cytometry. (C) The protein expressions of CDKN1B, Cyclin E1, CDK2 and SATB2 in hFOB1.19 cells were detected by western blot. (D, E, F, G) The relative expressions of CDKN1B, SATB2, CDK2 and Cyclin E1 were quantified via normalizing to β-actin. **P<0.01 compared to control. ^##^P<0.01 compared to 1 μM DEX. ^P<0.05, ^^P<0.01 compared to DEX + siRNA2.

**Fig 6:**
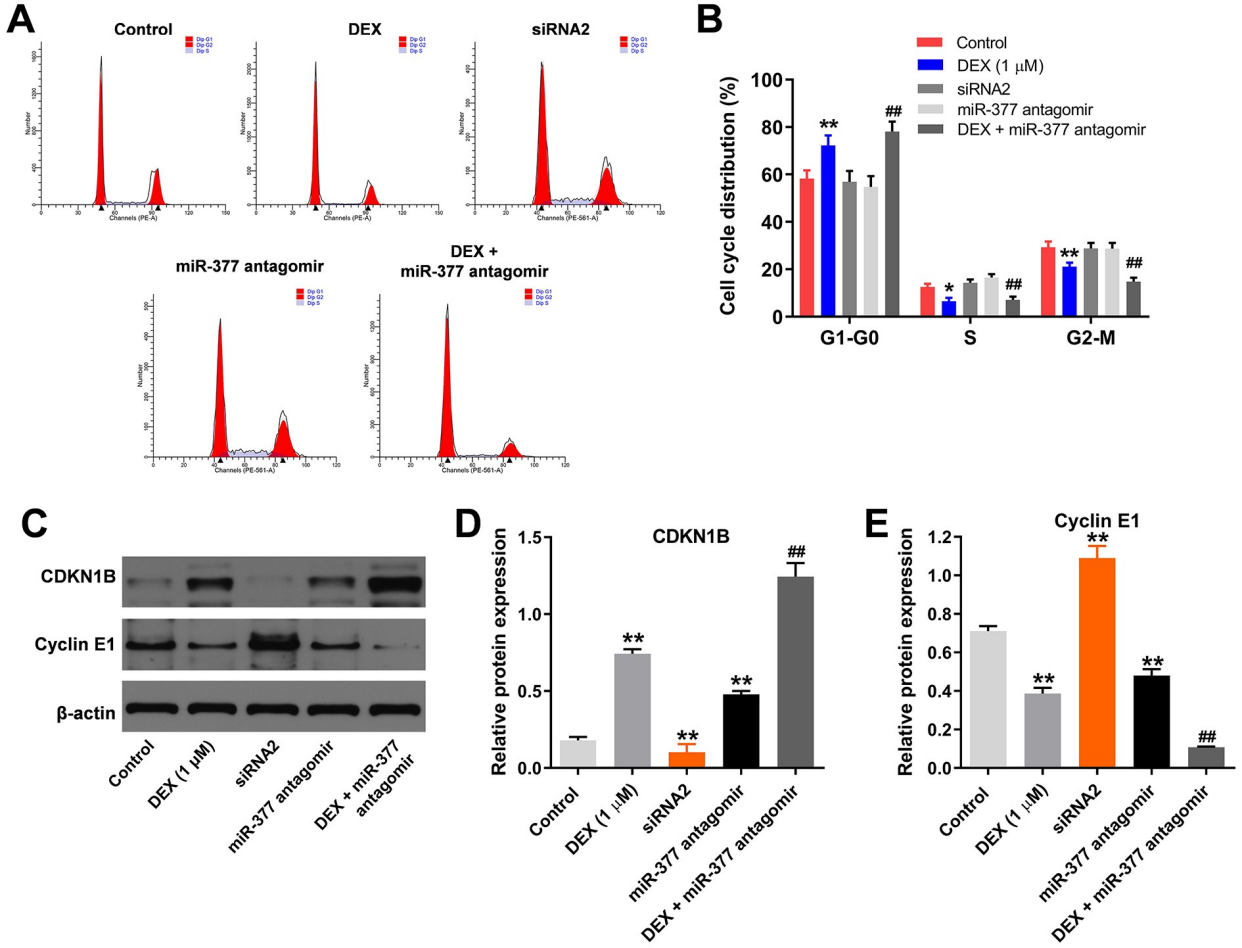
MiR-377 antagomir further aggravated DEX-induced G1 arrest in hFOB1.19 cells. (A, B) Cell cycle distribution was tested by flow cytometry. (C) The protein expressions of CDKN1B and Cyclin E1 in hFOB1.19 cells were detected by western blot. (D, E) The relative expressions of CDKN1B and Cyclin E1 were quantified via normalizing to β-actin. **P<0.01 compared to control. ^##^P<0.01 compared to 1 μM DEX.

To assess the cell cycle, flow cytometry was used. As shown in Fig 5A and 5B, DEX-induced G_1_ arrest in hFOB1.19 cells was significantly inhibited by silencing hsa_circ_0001275, while the effects of hsa_circ_0001275 siRNA2 on cell cycle distribution were significantly reversed in the presence of miR-377 antagomir. In addition, DEX-induced increase of CDKN1B and SATB2 expression was significantly inhibited by hsa_circ_0001275 knockdown (Fig 5C–5E). In contrast to these findings, the protein levels of CDK2 and Cyclin E1 in hFOB1.19 cells were significantly inhibited by DEX and this effect was obviously reversed in the presence of hsa_circ_0001275 siRNA2 (Fig 5C, 5F and 5G). Furthermore, the effects of hsa_circ_0001275 siRNA2 on these proteins were markedly reversed by miR-377 antagomir (Fig 5C–5G). On the other hand, miR-377 antagomir could aggravate DEX-induced G1 arrest in hFOB1.19 cells, while hsa_circ_0001275 siRNA2 or miR-377 antagomir alone did not affect the cell cycle distribution (Fig 6A and 6B). hsa_circ_0001275 siRNA2 significantly inhibited the expression of CDKN1B and increased the level of cyclin E1 (Fig 6C–6E). In contrast, miR-377 antagomir notably increased the level of CDKN1B and decreased the expression of cyclin E1 (Fig 6C–6E). Besides, the effect of DEX on these two proteins was further aggravated by miR-377 antagomir (Fig 6C–6E). In summary, miR-377 antagomir significantly reversed the effects of hsa_circ_0001275 siRNA2 on cell cycle distribution.

### CDKN1B overexpression notably reverses the effect of hsa_circ_0001275 siRNA2 on CDKN1B, Cyclin E1, CDK2 and SATB2

**Fig 7:**
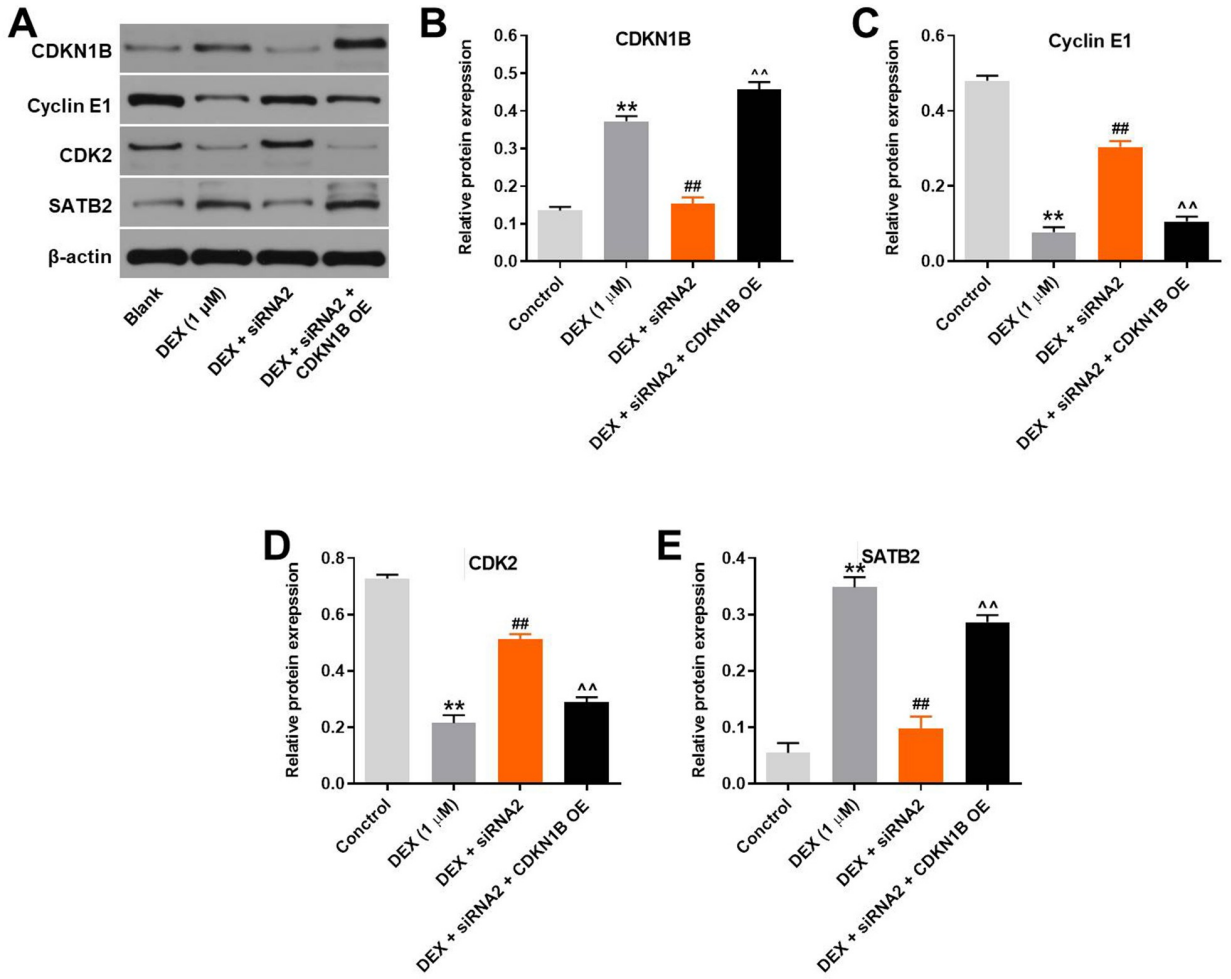
CDKN1B overexpression notably reverses the effect of hsa_circ_0001275 siRNA2 on CDKN1B, Cyclin E1, CDK2 and SATB2. (A) hFOB1.19 cells were treated with 1 μM DEX, DEX + hsa_circ_0001275 siRNA2 or DEX + hsa_circ_0001275 siRNA2 + pcDNA3.1-CDKN1B. Then, the protein expressions of CDKN1B, Cyclin E1, CDK2 and SATB2 in hFOB1.19 cells were detected by western blot. (B, C, D, E) The relative expressions of CDKN1B, SATB2, Cyclin E1 and CDK2 were quantified via normalizing to β-actin. **P<0.01 compared to control. ^##^P<0.01 compared to 1 μM DEX. ^^P<0.01 compared to DEX + siRNA2.

To further confirm the correlation between hsa_circ_0001275 and CDKN1B in hFOB1.19 cells, western blot was used. As shown in Fig 7A–7E, DEX significantly upregulated the expression of CDKN1B and SATB2, while this phenomenon was notably reversed by hsa_circ_0001275 knockdown. In contrast, DEX-induced decrease of cyclin E1 and CDK2 expressions was greatly reversed in the presence of hsa_circ_0001275 siRNA2. Meanwhile, the effect of hsa_circ_0001275 siRNA on these proteins was significantly reversed by overexpression of CDKN1B (Fig 7A–7E). Altogether, CDKN1B overexpression notably reverses the effect of hsa_circ_0001275 siRNA2 on CDKN1B, Cyclin E1, CDK2 and SATB2.

## Discussion

It has been reported that hsa_circ_0001275 is upregulated in osteoporosis [16]. In the present study, knockdown of hsa_circ_0001275 reversed DEX-induced osteoblast growth inhibition. The present study firstly explored the function of hsa_circ_0001275 in DEX-induced osteoblasts, and found that hsa_circ_0001275 could act as a potential target for the treatment of osteoporosis.

In the current study, hsa_circ_0001275 could sponge miR-377 in hFOB1.19 cells. Previous reports have confirmed that miR-377 participates in the development of various types of diseases [30, 31]. In addition, miR-377 was reported to be involved in the dysfunction of osteoblasts [32, 33]. The present study demonstrated that miR-377 downregulation reversed the effect of hsa_circ_0001275 siRNA on DEX-treated osteoblasts, supporting the biological function of miR-377 in osteoporosis. Meanwhile, some studies have indicated that other miRNAs might play roles in osteoporosis. For example, miR-494 was upregulated in bone formation [34]; miR-515-3p could be involved in translation regulation of osteoblasts [35]. Thus, more miRNAs involved in the progression of osteoporosis are needed to be explored.

CDKN1B (p27 Kip1) was targeted by miR-377. CDKN1B has been reported to regulate G_1_ progression and maintain the cell function in response to cell proliferation inhibition or cell differentiation [36, 37]. In addition, it has been confirmed that CDKN1B can act as a key modulator in cell growth by induction of CDK2 and Cyclin E1 [38, 39]. The findings were consistent to these of previous studies. Meanwhile, Wang *et al* [40] demonstrated that miR-377 induced G_1_ arrest in glioma cells via targeting PTEN. The present study reported similar data to those published previously. PTEN has been shown to regulate G_1_ phase distribution in cells [41]. Similar function was noted between CDKN1B and PTEN. On the other hand, more mRNAs can be targeted by miR-377 [42, 43]. Zhou W *et al* found NR6A1 was targeted by miR-377 in gastric cancer [43], and miR-377 could alleviate myocardial injury induced by Hypoxia/Reoxygenation via downregulating LILRB2 expression [31]. Therefore, more mRNAs regulated by miR-377 in osteoblasts need to be identified in the future.

Meanwhile, our study indicated that hsa_circ_0001275 did not affect the expression of miR-377. In ceRNA network, circRNA could exert its biological function through binding with miRNA, and this phenomenon could decrease the biological function of miRNA [44]. Thus, our data further confirmed that hsa_circ_0001275 could sponge miR-377.

The present study contains certain limitations. The current investigation focused only on the association between hsa_circ_0001275 and miR-377. In addition, only one target of miR-377 was identified. Furthermore, the relation between SATB2 and cell cycle progression in osteoblasts needs to be further explored. Moreover, the effect of Dex+miR377 antagomir on CDK2 and STAB2 levels remains unclear. Therefore, additional studies are required in the future to confirm these findings.

In conclusion, knockdown of hsa_circ_0001275 reversed DEX-induced osteoblast growth inhibition via activation of the miR-377/CDKN1B axis. Therefore, our finding might shed new lights on the treatment of osteoporosis.

## Supporting information

S1 File (ZIP)
