# Supplementary figures and images for: Knockdown of hsa_circ_0001275 reverses dexamethasone-induced osteoblast growth inhibition via mediation of miR-377/CDKN1B axis
Source: PLoS One. 2021 May 27;16(5):e0252126. doi: 10.1371/journal.pone.0252126 (PMC8158950; doi:10.1371/journal.pone.0252126)

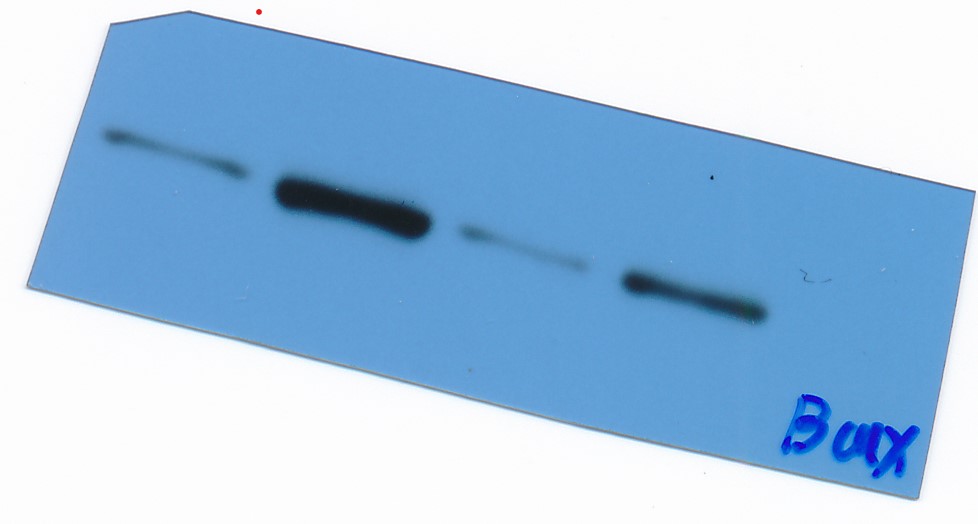

Supplement: S1 File — (ZIP) [file pone.0252126.s001.zip › western blot raw data/Figure 2 Bax-21KD.jpg]

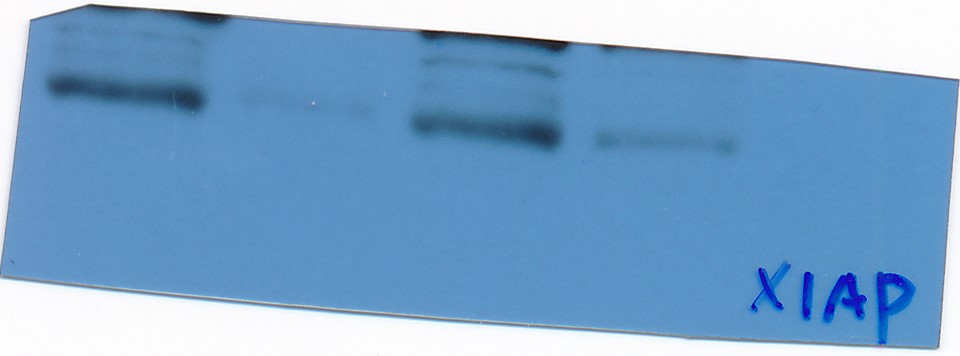

Supplement: S1 File — (ZIP) [file pone.0252126.s001.zip › western blot raw data/Figure 2 XIAP-56 KD.jpg]

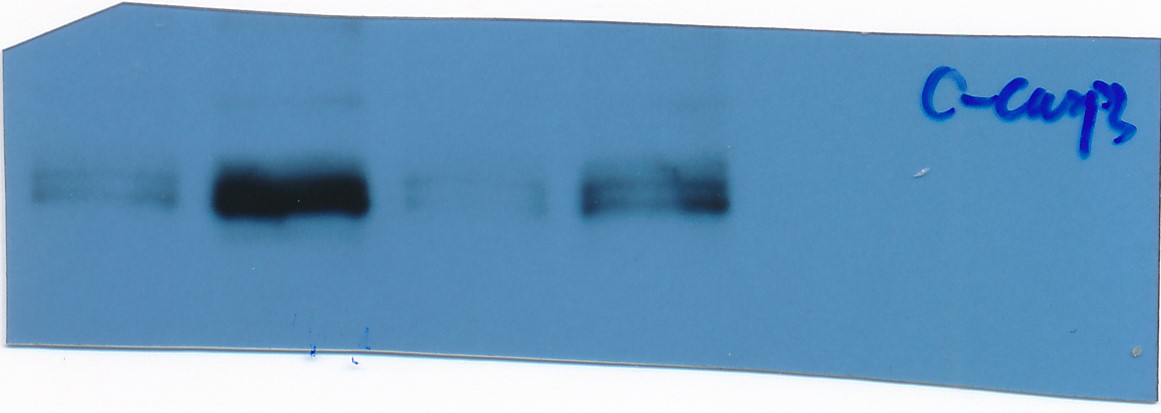

Supplement: S1 File — (ZIP) [file pone.0252126.s001.zip › western blot raw data/Figure 2 cleaved caspase 3-17 KD.jpg]

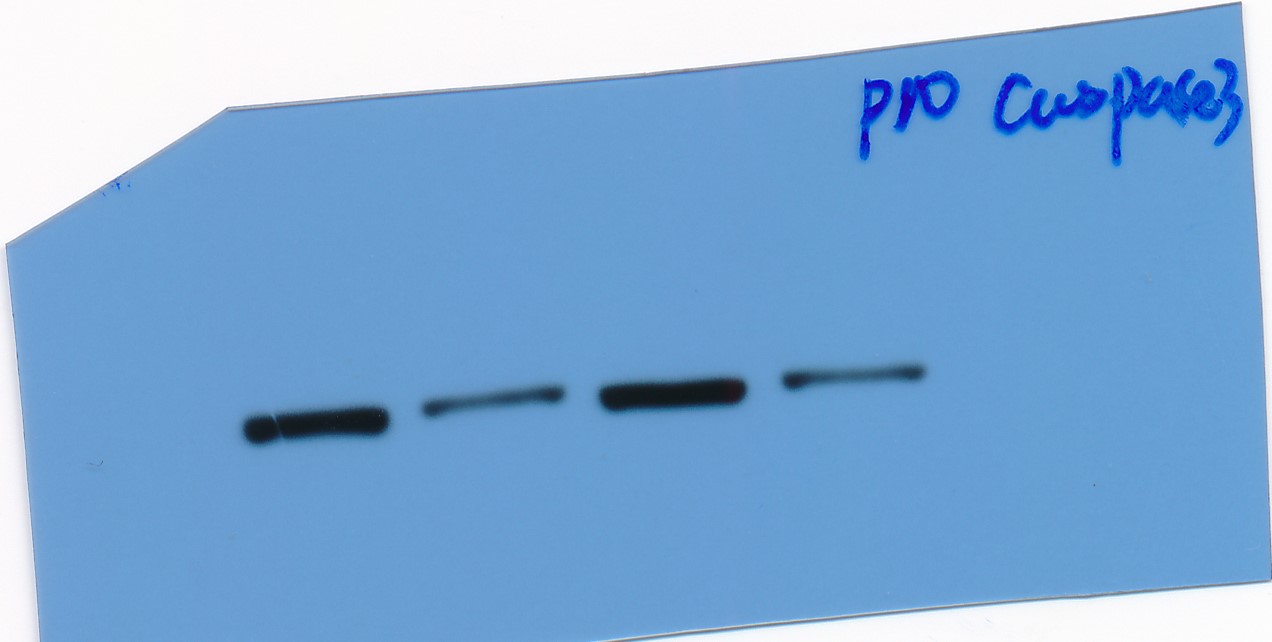

Supplement: S1 File — (ZIP) [file pone.0252126.s001.zip › western blot raw data/Figure 2 pro-caspase 3-32 KD.jpg]

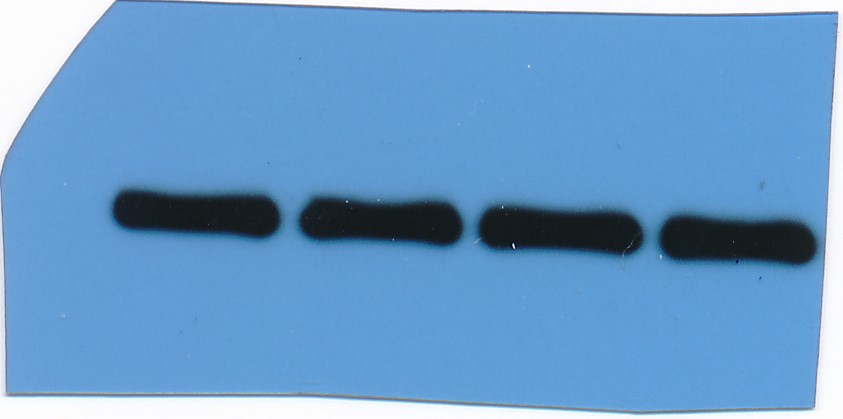

Supplement: S1 File — (ZIP) [file pone.0252126.s001.zip › western blot raw data/Figure 2 a┬-actin-43 KD.jpg]

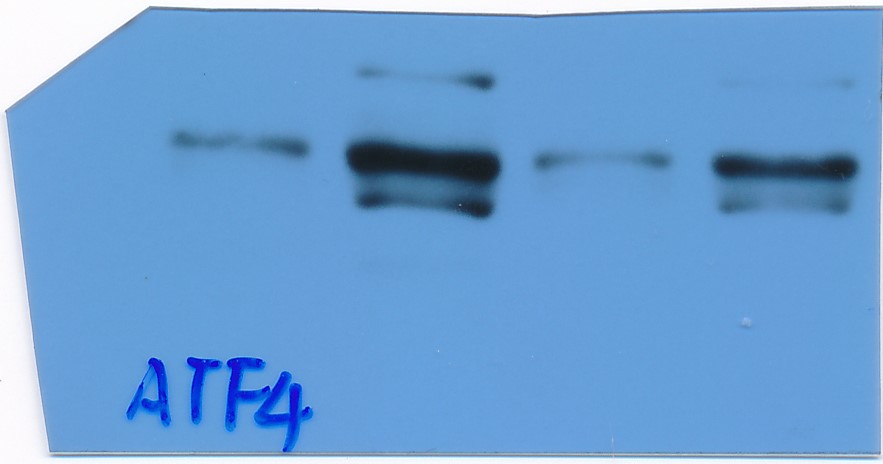

Supplement: S1 File — (ZIP) [file pone.0252126.s001.zip › western blot raw data/Figure 3 ATF4-38 KD.jpg]

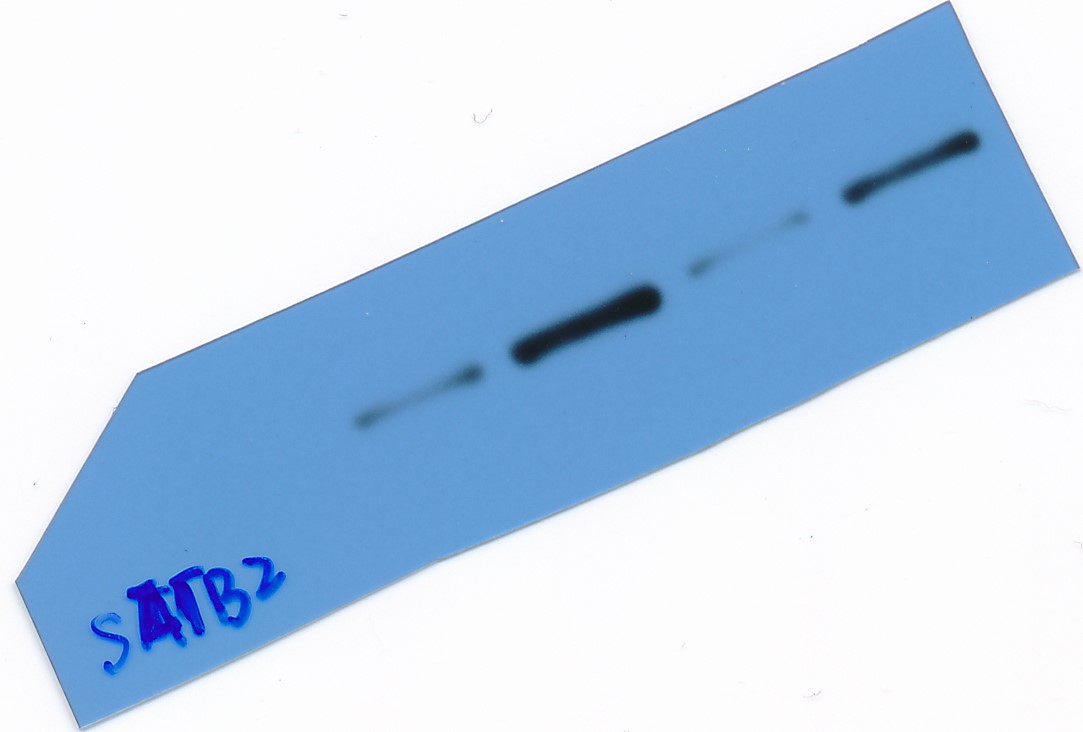

Supplement: S1 File — (ZIP) [file pone.0252126.s001.zip › western blot raw data/Figure 3 SATB2-81KD.jpg]

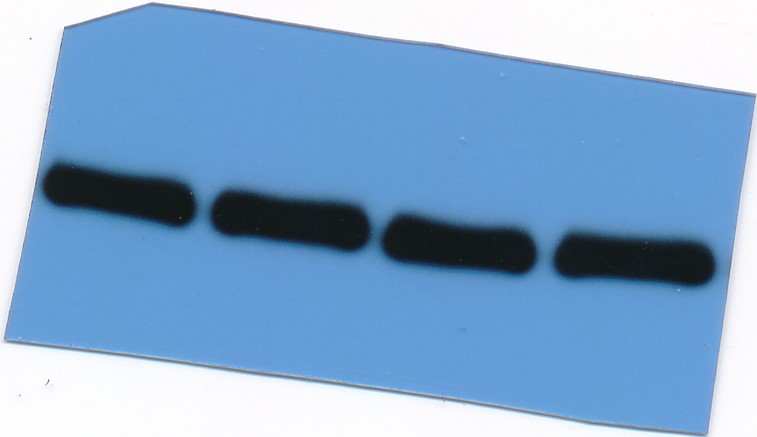

Supplement: S1 File — (ZIP) [file pone.0252126.s001.zip › western blot raw data/Figure 3 a┬-actin-43 KD.jpg]

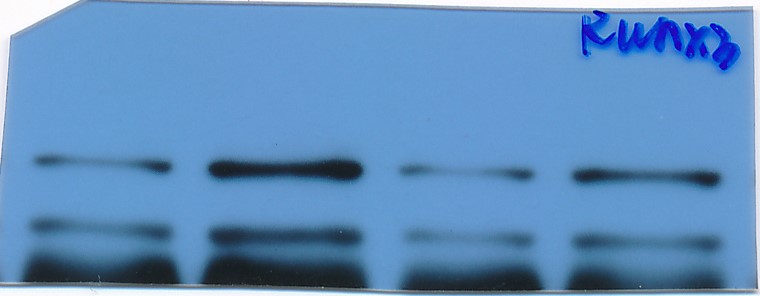

Supplement: S1 File — (ZIP) [file pone.0252126.s001.zip › western blot raw data/Figure 3-Runx3-46 KD.jpg]

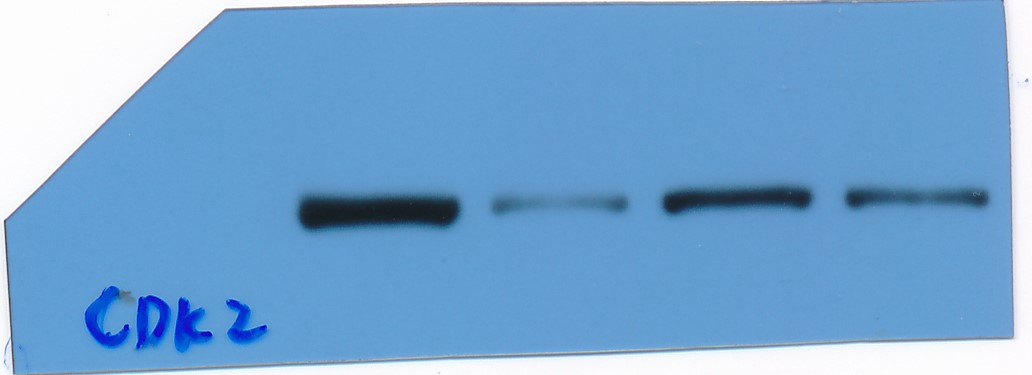

Supplement: S1 File — (ZIP) [file pone.0252126.s001.zip › western blot raw data/Figure 5 CDK2-34 KD.jpg]

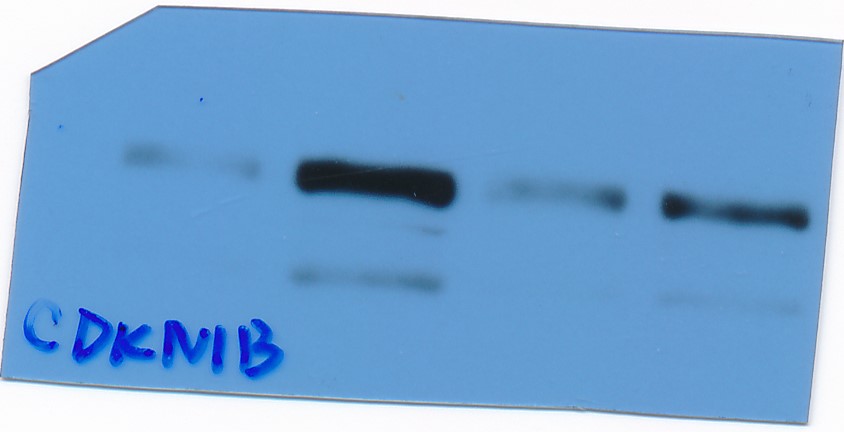

Supplement: S1 File — (ZIP) [file pone.0252126.s001.zip › western blot raw data/Figure 5 CDKN1B-22 KD.jpg]

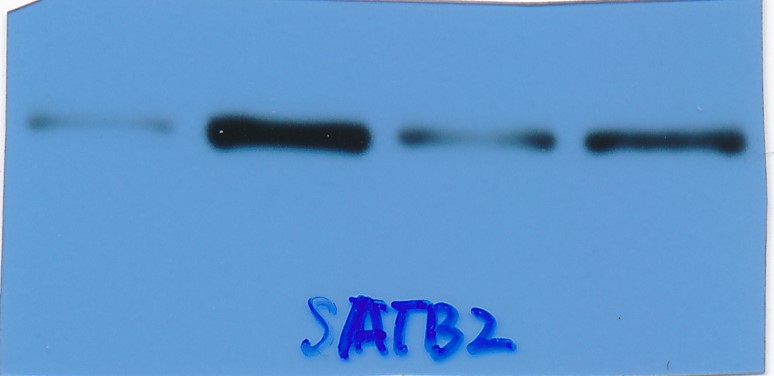

Supplement: S1 File — (ZIP) [file pone.0252126.s001.zip › western blot raw data/Figure 5 SATB2-81KD.jpg]

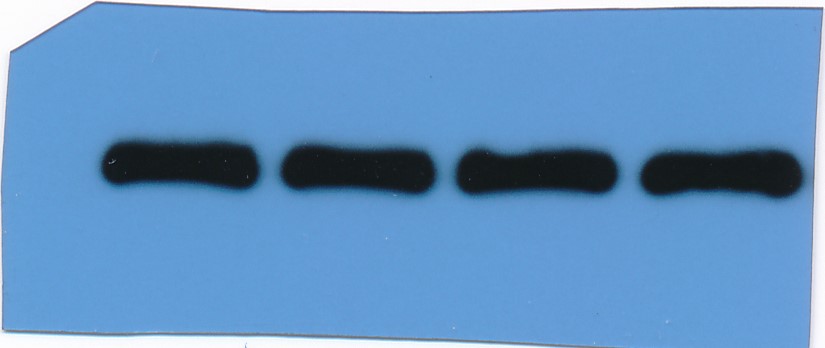

Supplement: S1 File — (ZIP) [file pone.0252126.s001.zip › western blot raw data/Figure 5 a┬-actin-43 KD.jpg]

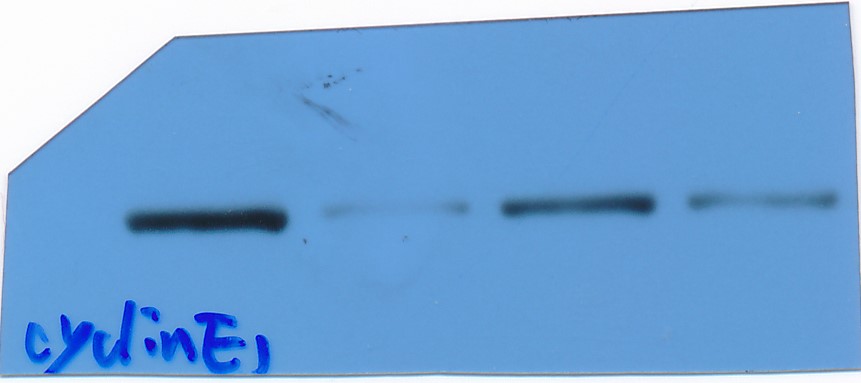

Supplement: S1 File — (ZIP) [file pone.0252126.s001.zip › western blot raw data/Figure 5-cyclin E1-47 KD.jpg]

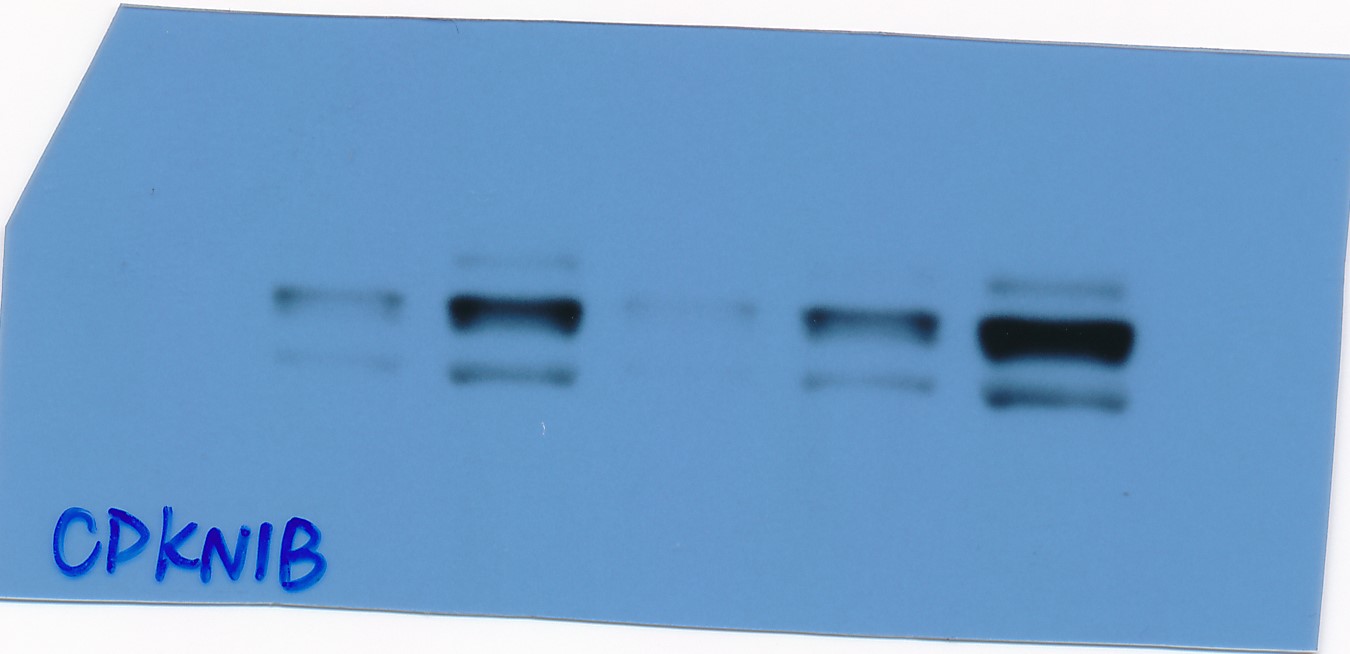

Supplement: S1 File — (ZIP) [file pone.0252126.s001.zip › western blot raw data/Figure 6 CDKN1B-22 KD.jpg]

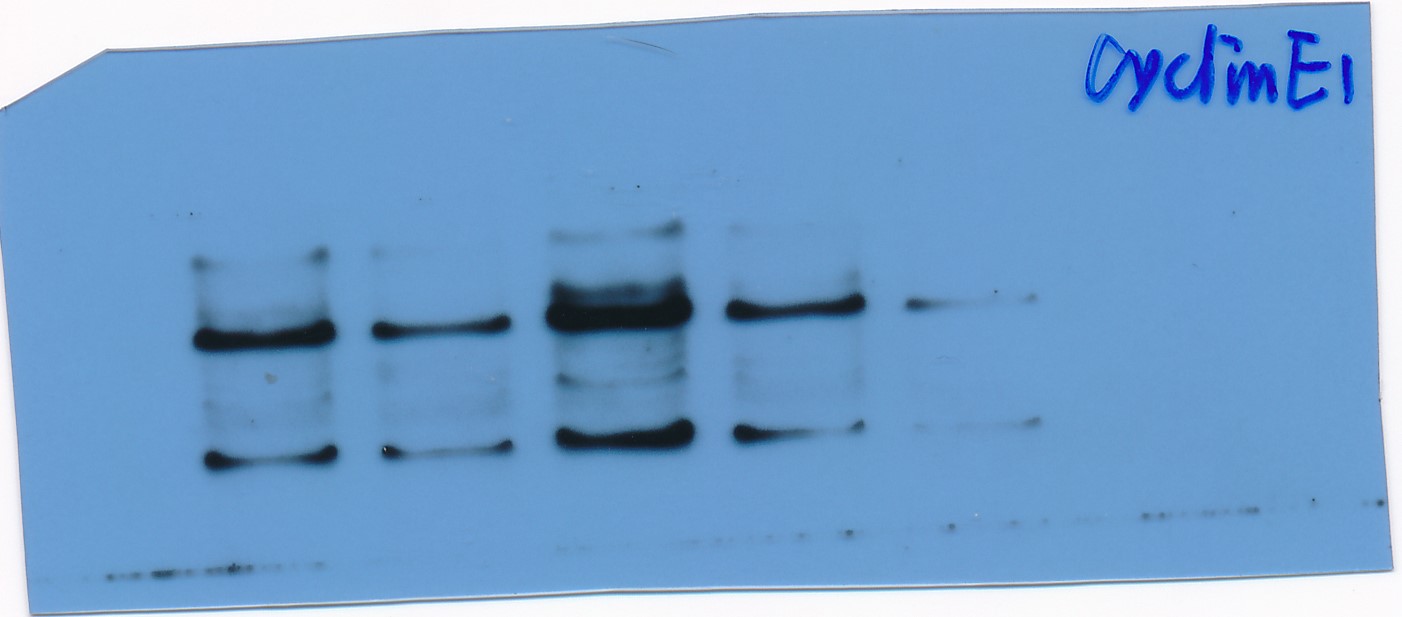

Supplement: S1 File — (ZIP) [file pone.0252126.s001.zip › western blot raw data/Figure 6 cyclin E1-47 KD.jpg]

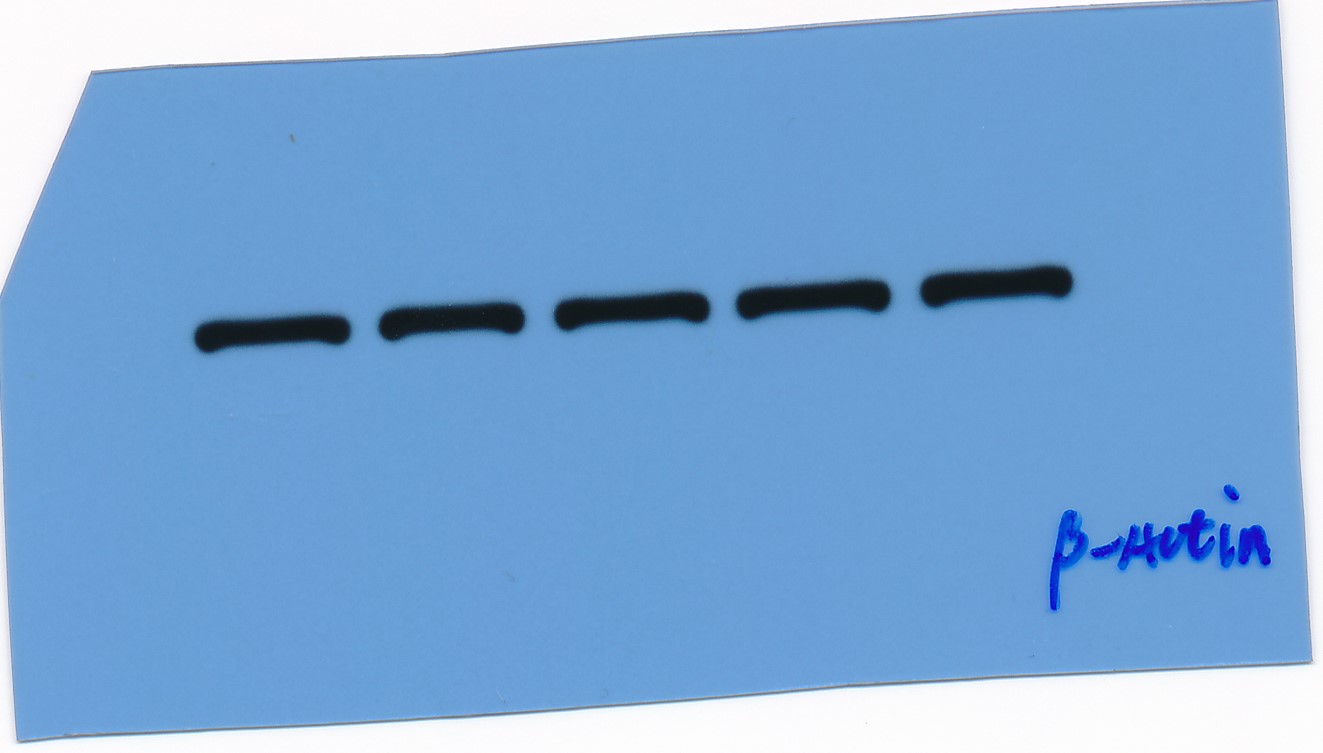

Supplement: S1 File — (ZIP) [file pone.0252126.s001.zip › western blot raw data/Figure 6-a┬-actin-43KD.jpg]

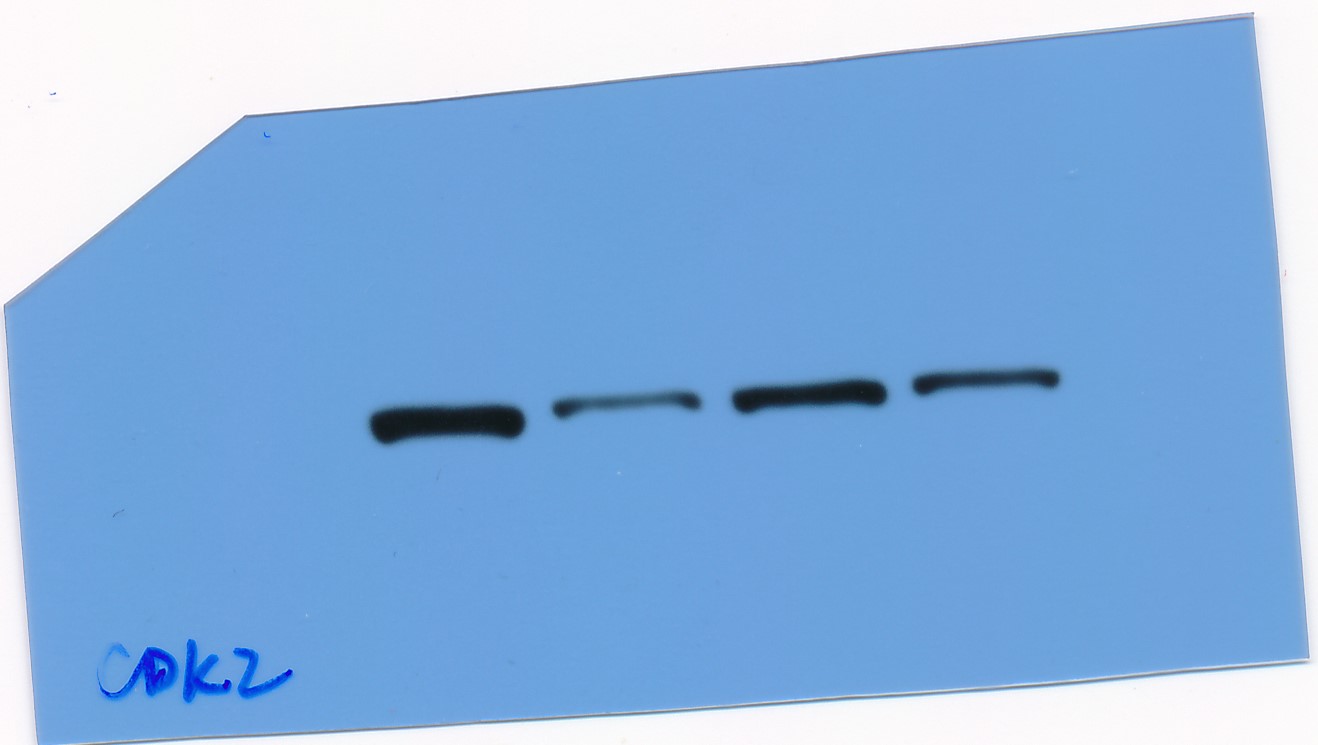

Supplement: S1 File — (ZIP) [file pone.0252126.s001.zip › western blot raw data/Figure 7 CDK2-34 KD.jpg]

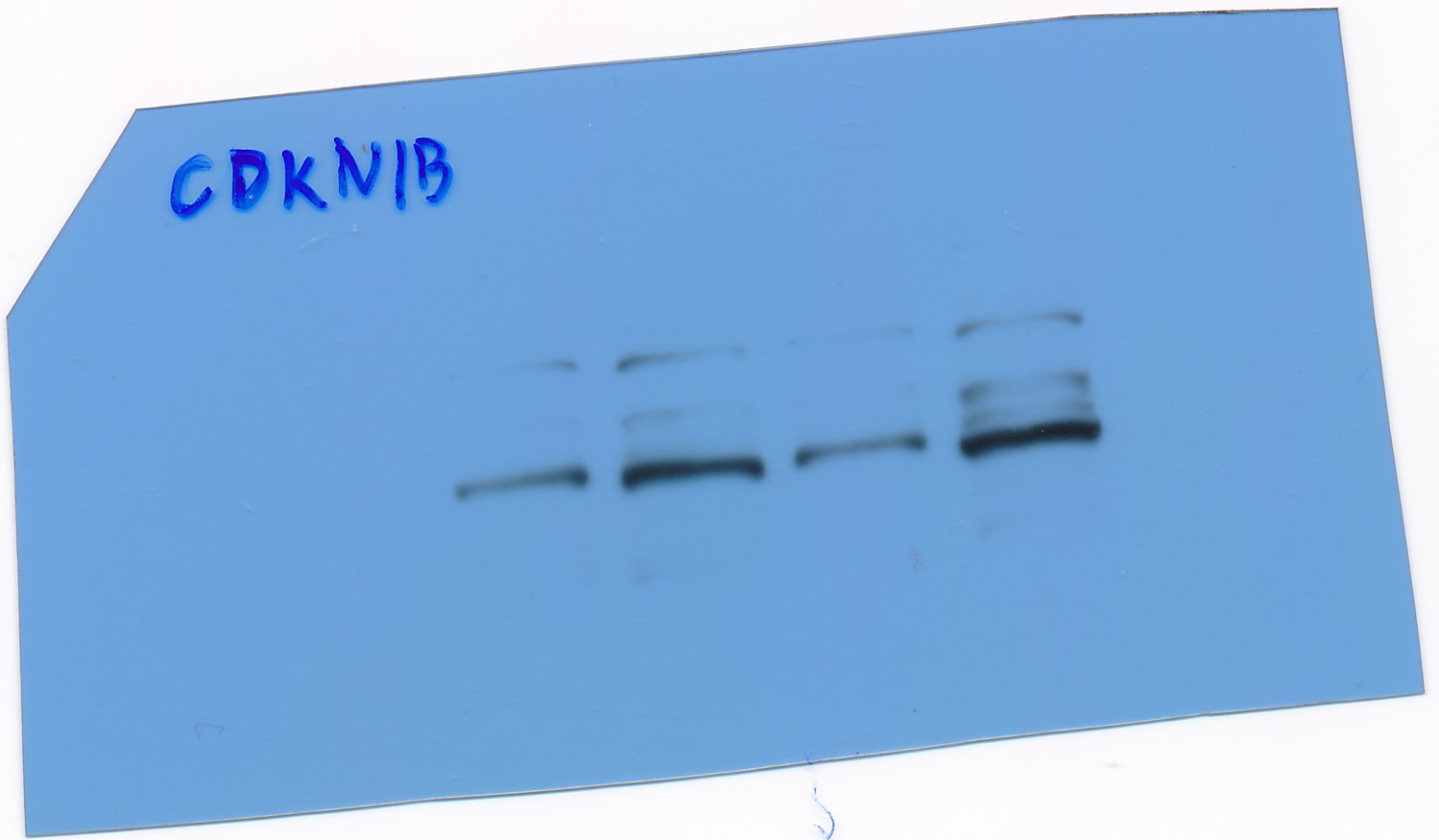

Supplement: S1 File — (ZIP) [file pone.0252126.s001.zip › western blot raw data/Figure 7 CDKN1B-22 KD.jpg]

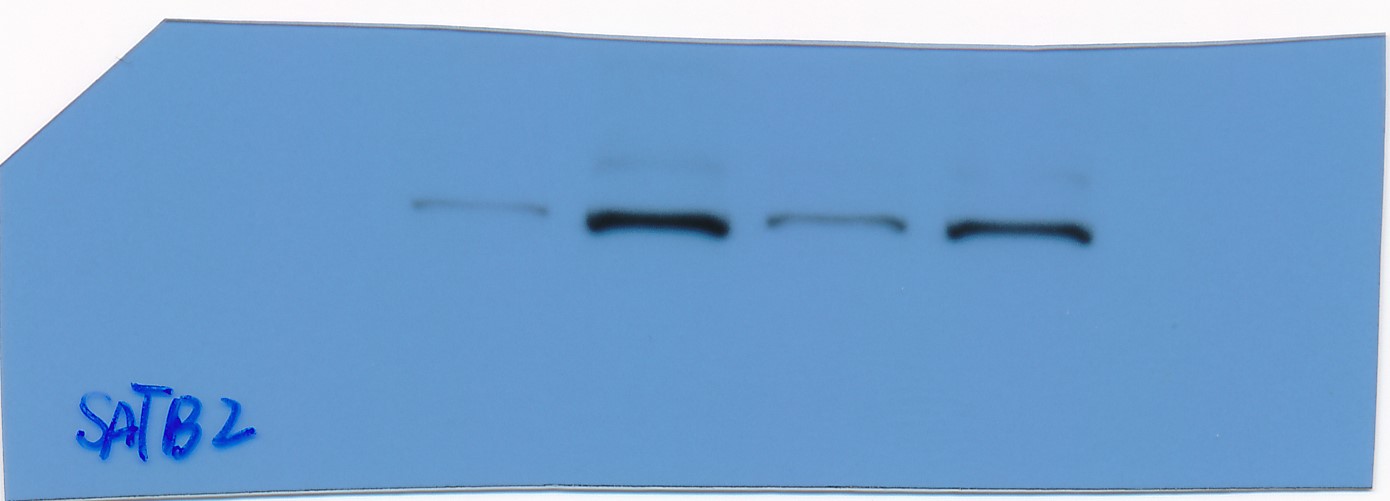

Supplement: S1 File — (ZIP) [file pone.0252126.s001.zip › western blot raw data/Figure 7 SATB2-81 KD.jpg]

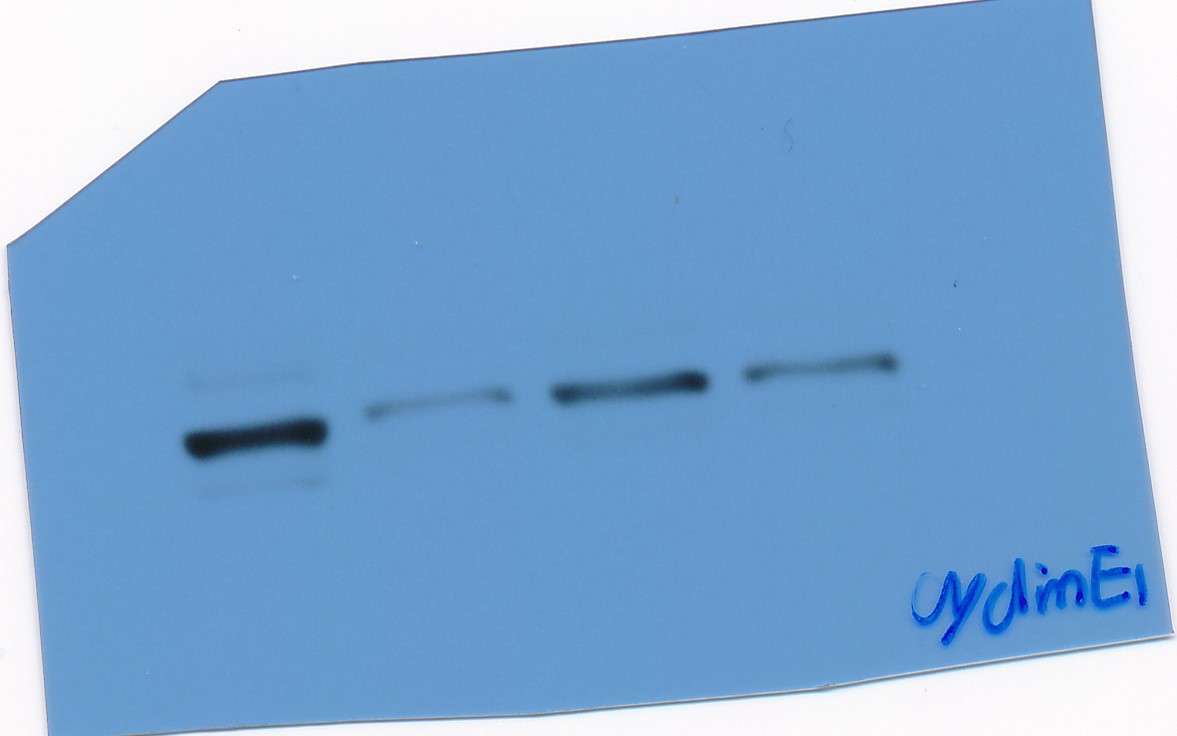

Supplement: S1 File — (ZIP) [file pone.0252126.s001.zip › western blot raw data/Figure 7 cyclin E1-47 KD.jpg]

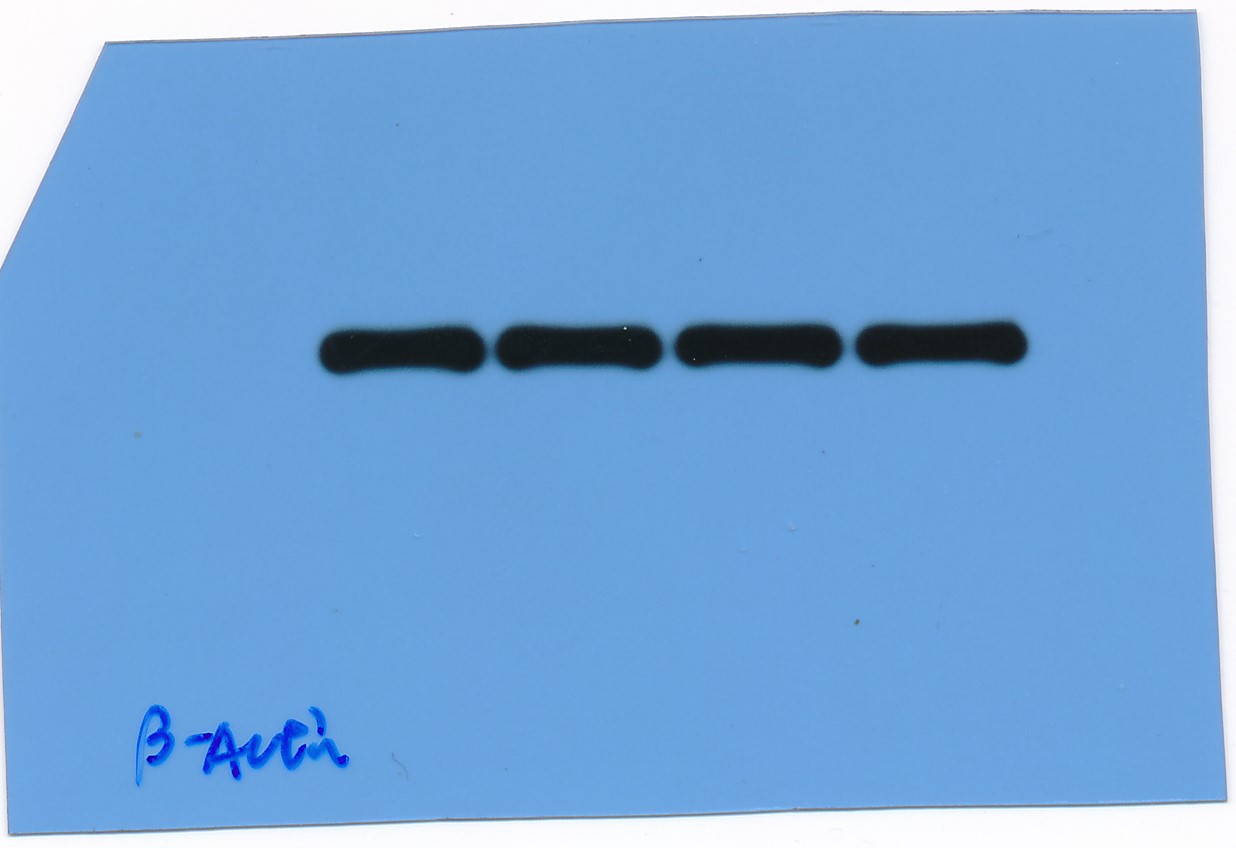

Supplement: S1 File — (ZIP) [file pone.0252126.s001.zip › western blot raw data/Figure 7 a┬-actin-43 KD.jpg]
